# Carvone Decreases Melanin Content by Inhibiting Melanoma Cell Proliferation via the Cyclic Adenosine Monophosphate (cAMP) Pathway

**DOI:** 10.3390/molecules25215191

**Published:** 2020-11-07

**Authors:** Wesuk Kang, Dabin Choi, Soyoon Park, Taesun Park

**Affiliations:** Department of Food and Nutrition, BK21 FOUR, College of Human Ecology, Yonsei University, 50 Yonsei-ro, Seodaemun-gu, Seoul 03722, Korea; wesuk42@naver.com (W.K.); vin1411@naver.com (D.C.); thdbs1201@naver.com (S.P.)

**Keywords:** carvone, melanoma cell, cell proliferation, cAMP, melanin

## Abstract

Melanin, which determines the color of the skin and hair, is initially synthesized to protect the skin from ultraviolet light; however, excessive melanin pigmentation caused by abnormal cell proliferation can result in various melanocytic lesions. Cyclic adenosine monophosphate (cAMP) is known to regulate cell cycle progression and consequently to inhibit the division of abnormally proliferating cells. In this work, we aimed to test whether carvone, a scent compound from plants, inhibits proliferation and subsequently reduces melanin content of melanoma cells and to determine whether its beneficial effects are mediated by the cAMP pathway. We found that carvone decreases melanin content and inhibits melanoma cell proliferation in a concentration-dependent manner. Meanwhile, it inhibited the activation of cell cycle-associated proteins such as cyclin-dependent kinase 1 (CDK1). Of note, the beneficial effects of carvone were abrogated by cAMP inhibition. Our findings indicate potential benefits of carvone for the treatment of melanomas and presumably other hyperpigmentation-related dermatological disorders such as melasmas, lentigines, and excessive freckles.

## 1. Introduction

Melanin, the end product of melanogenesis, is produced exclusively in melanocytes and determines the color of human skin, eyes, and hair. Melanin is initially synthesized to protect the skin from ultraviolet light, but overproduction and accumulation of melanin may lead to numerous hyperpigmentation-related dermatological disorders, including melasmas, lentigines (age spots), excessive freckles, nevi, and melanomas [1,2,3]. The major contributing factor of these melanocytic lesions is the loss of control of cell proliferation owing to ultraviolet-induced damage and other environmental factors, such as stress [3,4,5,6,7].

Cyclic adenosine monophosphate (cAMP), acting as an intracellular secondary messenger, is widely accepted as a key mediator of diverse biological processes [8,9]. Particularly, it is well known that cAMP regulates cell cycle progression: In many tumor cells with high proliferation rates, cAMP is a negative regulator (secondary messenger) of proliferation, with lower basal cAMP levels in most tumor cells than those in normal cells [10,11,12,13]. For example, in melanoma cells, the elevation of cAMP concentrations by treatment with forskolin (an adenylyl cyclase (ADCY) activator) delays cell cycle progression and cell proliferation [14]. Furthermore, other cAMP-upregulating agents such as 8-bromo-cAMP (a cAMP analog) and erythro-9-(2-hydroxy-3-nonyl) adenine (a cAMP phosphodiesterase 2 inhibitor) have been shown to inhibit melanoma cell growth [15].

We recently conducted a phenotype-based approach to screening for compounds in B16F10 melanoma cells to find small-molecule inhibitors of melanin synthesis [16,17]. By this method, we found that carvone treatment markedly decreases melanin content. Carvone is a scent compound categorized as a terpenoid (with the formula C_10_H_14_O) and is found in various plants, mostly in caraway, spearmint, and dill [18,19]. Carvone has been designated as a generally recognized as safe (GRAS) substance for application as a flavoring agent in the food industry. This compound is well known for its antioxidant, antimicrobial, hypotensive, and hypolipidemic effects [20,21,22]. In the present study, we sought to examine whether carvone decreases melanin content by inhibiting the proliferation of melanoma cells, and if so, to explore whether carvone exerts its antiproliferative effect through the cAMP pathway.

## 2. Results

### 2.1. Carvone Decreases Melanin Content of B16F10 Melanoma Cells

**Figure 1:**
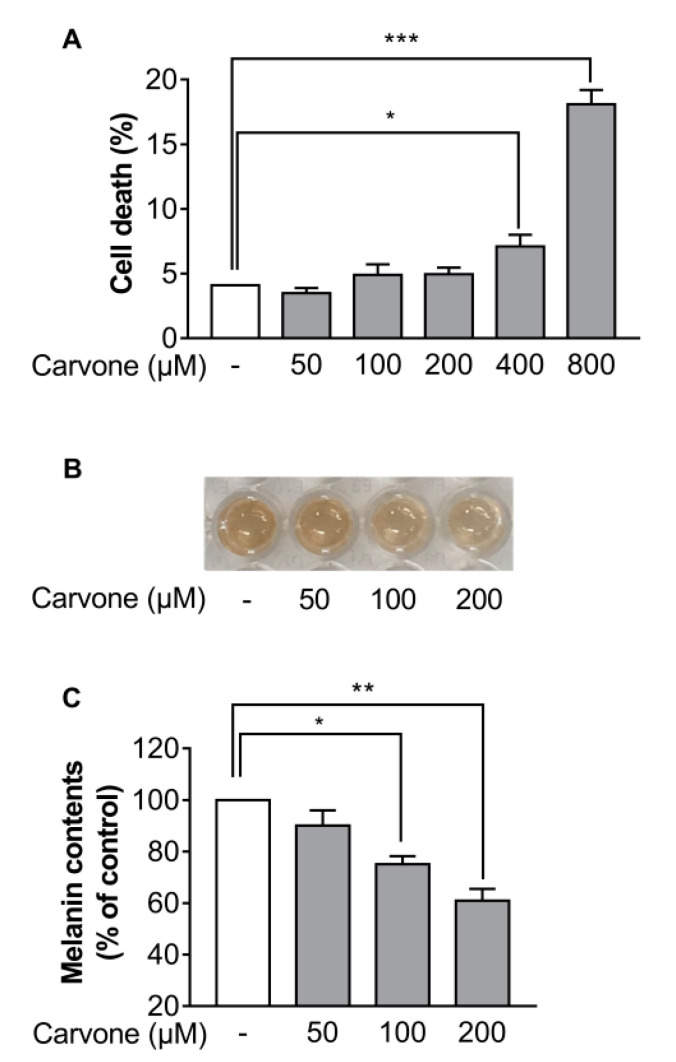
Carvone reduces melanin content of B16F10 melanoma cells. Cells were cultured with either various concentrations of carvone or vehicle 48 h. (**A**) The percentage of dead cells was assessed by trypan blue exclusion assay. (**B**,**C**) The melanin production was visualized and quantified. The data are shown as the mean ± standard error of the mean (SEM) of three experiments; * *p* < 0.05, ** *p* < 0.01, *** *p* < 0.001.

Firstly, to investigate the effect of carvone on cell death, the B16F10 melanoma cells were treated with either vehicle (dimethyl sulfoxide (DMSO)) or various concentrations of carvone (50–800 µM). Cell death was significantly increased after treatment with above 200 µM carvone (Figure 1A). Therefore, the maximum concentration of carvone used in the following experiments did not exceed 200 µM. Next, to determine whether carvone affects melanin content, we measured melanin content of B16F10 melanoma cells treated with either vehicle (DMSO) or three concentrations of carvone (50, 100, or 200 µM). We found that carvone decreased melanin content in a concentration-dependent manner (Figure 1B,C).

### 2.2. Carvone Suppresses B16F10 Melanoma Cell Proliferation

**Figure 2:**
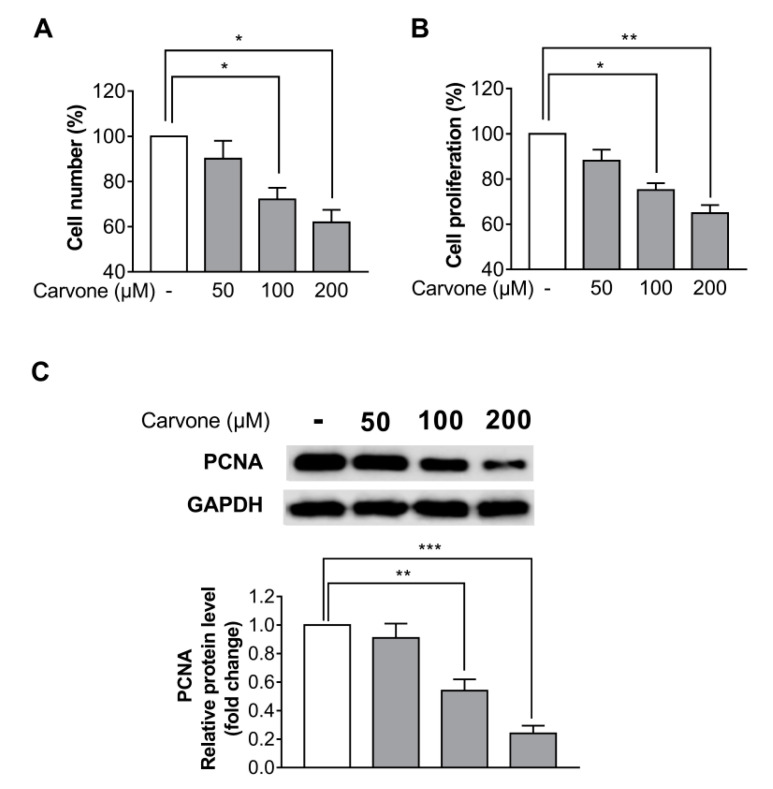
Carvone suppresses B16F10 melanoma cell proliferation. The cells were cultured with carvone at various concentrations (0, 50, 100, or 200 µM) for 48 h. Cell proliferation rates were determined by (**A**) cell counting and (**B**) the 3-(4,5-dimethylthiazol-2-yl)-2,5-diphenyltetrazolium bromide (MTT) assay. (**C**) The protein expression of proliferating cell nuclear antigen (PCNA) was evaluated after incubation with the indicated concentrations of carvone for 48 h. The data are shown as the mean ± SEM of three experiments; * *p* < 0.05, ** *p* < 0.01, *** *p* < 0.001.

To investigate the effects of carvone on cell proliferation, B16F10 melanoma cells were treated with either vehicle or carvone at different concentrations (50, 100, or 200 µM) and cultured for 48 h. Both the cell counting and a 3-(4,5-dimethylthiazol-2-yl)-2,5-diphenyltetrazolium bromide (MTT) assay were carried out to measure the cell proliferation. The cell proliferation was significantly inhibited by 100 and 200 µM carvone (Figure 2A,B). Considering that melanin content normalized to relative cell numbers did not significantly differ between the control and carvone treatment (50, 100, or 200 µM; data not shown), the carvone-induced melanin content reduction is likely attributed to decreased cell proliferation. Furthermore, we analyzed the protein expression of a cell proliferation marker, proliferating cell nuclear antigen (PCNA). We found that carvone significantly decreased the expression of the PCNA protein in a dose-dependent manner (Figure 2C). Based on these findings, further experiments were conducted at the 200 µM carvone concentration. Carvone treatment had no influence on the proliferation of Hs68 dermal fibroblasts and HaCaT keratinocytes (Figure S1).

### 2.3. Carvone Activates the cAMP Pathway in B16F10 Melanoma Cells

**Figure 3:**
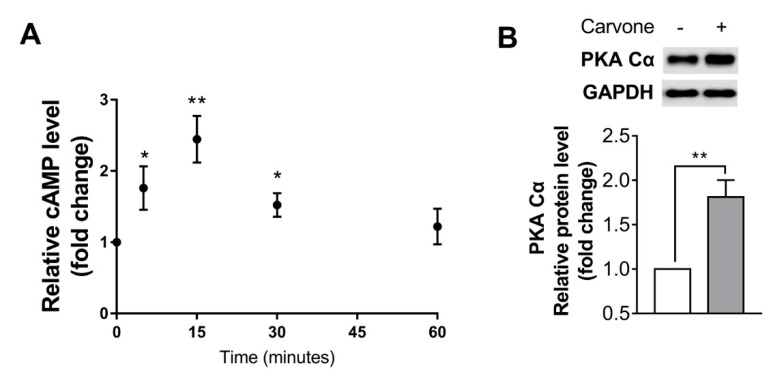
Carvone activates cyclic adenosine monophosphate (cAMP) signaling in B16F10 melanoma cells. (**A**) The time course of carvone-stimulated cAMP concentration was estimated after incubation with 200 μM carvone for 5, 15, 30, or 60 min. (**B**) The protein kinase A catalytic subunit (PKA Cα) protein expression was determined after treatment with either vehicle or 200 μM carvone for 30 min. The data are presented as the mean ± SEM of three experiments; * *p* < 0.05, ** *p* < 0.01.

In an effort to investigate the involvement of cAMP signaling in carvone’s effects on B16F10 melanoma cells, we evaluated concentrations of cAMP time-dependently after treatment with 200 µM carvone. A peak of cAMP concentrations was noted at 15 min, and the level returned to baseline within 60 min (Figure 3A). In addition, carvone markedly raised the protein kinase A catalytic subunit (PKA Cα) protein level, which could be upregulated by cAMP (Figure 3B).

### 2.4. Carvone Has No Effect on Melanogenesis-Related Gene Expression

**Figure 4:**
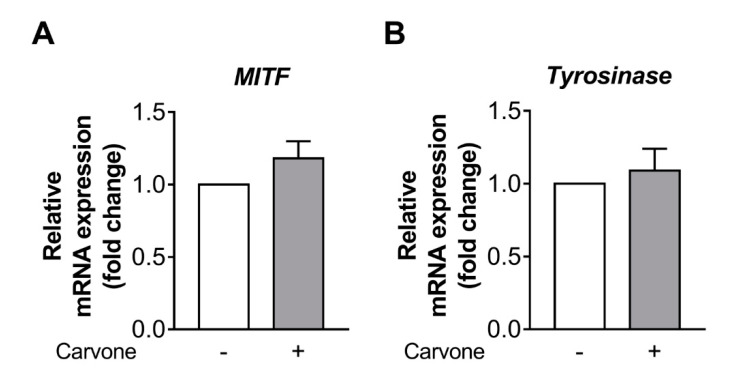
Carvone has no effect on melanogenesis-related gene expression in B16F10 melanoma cells. The cells were treated with either carvone (200 μM) or vehicle for 12 h. (**A**,**B**) The mRNA expression levels of microphthalmia-associated transcription factor (MITF) and tyrosinase were analyzed. The data are presented as the mean ± SEM of three experiments.

To understand the inhibitory mechanism of action of carvone on melanin synthesis, we analyzed the expression of melanogenesis-associated genes (tyrosinase and microphthalmia-associated transcription factor (MITF)) in B16F10 cells treated with either vehicle or carvone. We demonstrated that carvone had no effect on MITF and tyrosinase gene expression (Figure 4A,B).

### 2.5. Carvone Decreases Melanin Content through the cAMP Pathway

**Figure 5:**
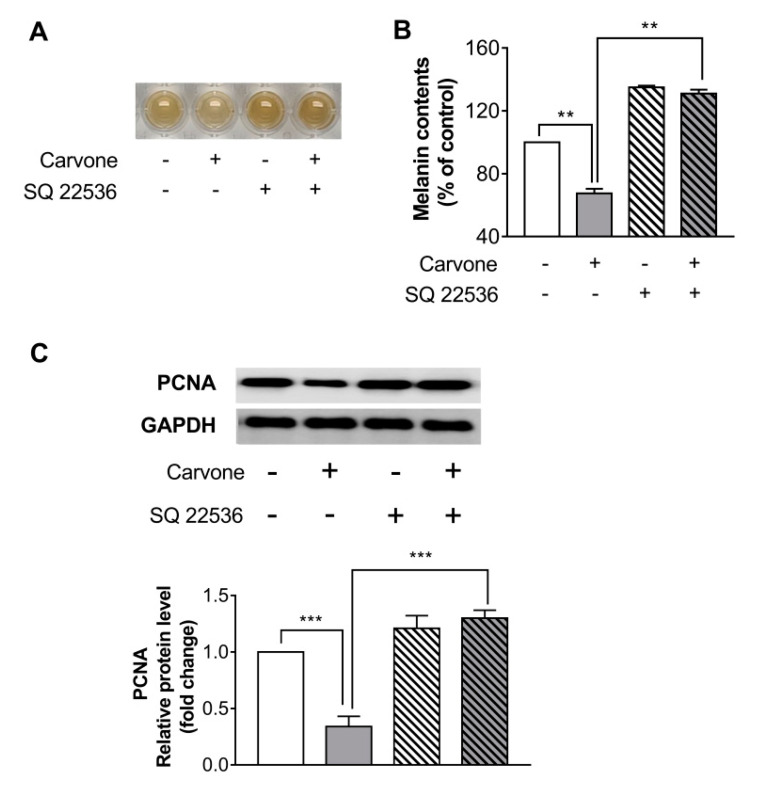
Carvone reduces melanin content and cell proliferation through the cAMP pathway. B16F10 melanoma cells were treated with either carvone (200 μM) or vehicle for 48 h. Either SQ22536 (50 μM) or vehicle was preincubated with the cells for 30 min before the carvone treatment. (**A**,**B**) Melanin content and (**C**) protein expression of PCNA were measured. The data are shown as the mean ± SEM of three experiments; ** *p* < 0.01, *** *p* < 0.001.

In an effort to further investigate whether the carvone-stimulated melanin content reduction is mediated by the cAMP signaling, the cells were preincubated with SQ22536 (50 µM) for 30 min before carvone treatment. The treatment with SQ22536 abrogated the carvone-driven melanin content reduction in B16F10 melanoma cells (Figure 5A,B). SQ22536 also significantly abolished the inhibitory effects of carvone on PCNA protein expression (Figure 5C).

### 2.6. Carvone Affects Cell Cycle-Related Protein Expression via the cAMP Signaling Pathway

**Figure 6:**
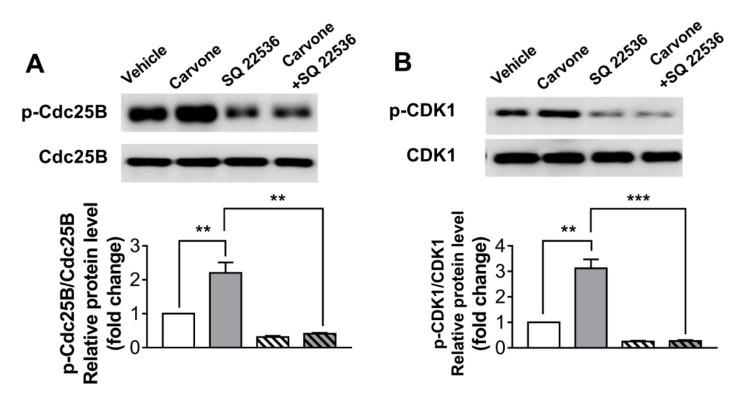
Carvone affects cell cycle-related protein expression via the cAMP pathway. B16F10 melanoma cells were cultured with either carvone (200 μM) or vehicle for 12 h. The vehicle or SQ22536 (50 μM) was preincubated with the cells for 30 min before carvone treatment. (**A**) Phosphorylation of cell division cycle 25B (Cdc25B) and (**B**) cyclin-dependent kinase 1 (CDK1) was assessed. The data are shown as the mean ± SEM of three experiments; ** *p* < 0.01, *** *p* < 0.001.

**Figure 7:**
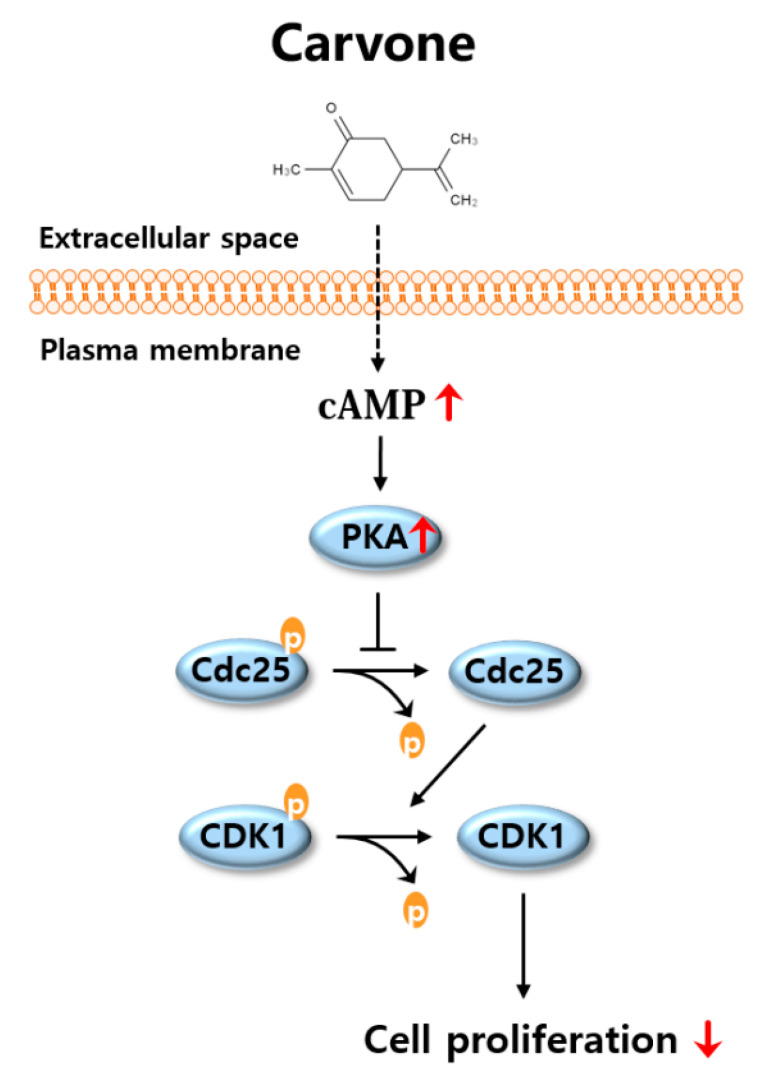
The proposed mechanism by which carvone decreases melanin content by inhibiting melanoma cell proliferation. As shown in a schematic illustration, the stimulation of the cAMP signaling pathway by carvone is responsible for the inhibition of cell proliferation. The downstream molecular mechanism by which cAMP-mediated inhibition of cell growth seems to be associated with the phosphorylation of Cdc25 and CDK1 proteins.

To explore whether carvone affects cell cycle progression, we tried to assess expression levels of cell cycle-related proteins in carvone-treated B16F10 melanoma cells. We demonstrated that the phosphorylation of cell division cycle 25B (Cdc25B) and cyclin-dependent kinase 1 (CDK1) was dramatically enhanced by carvone treatment. Consistently with the cell proliferation results, the SQ22536 pretreatment abrogated the carvone-induced phosphorylation of Cdc25B and CDK1 in B16F10 melanoma cells (Figure 6A,B). Considering that the beneficial effects of carvone were abrogated by cAMP inhibitor, the activation of the cAMP pathway by carvone is the possible mechanism for the inhibition of melanoma cell proliferation (Figure 7).

## 3. Discussion

It is widely known that mammalian cell proliferation is tightly regulated by a number of CDKs [23,24]. For example, CDK1 mediates the transition from the G2 phase to M phase, whereas CDK2 regulates the G1–S transition and S phase progression; CDKs 4 and 6 govern the progression from the G1 phase [25,26,27]. Notably, accumulating evidence suggests that cAMP signaling can strongly suppress the activity of CDK1 mainly owing to the phosphorylation of Cdc25B and subsequent phosphorylation of CDK1 in various cell types, including melanoma cells [14,28,29]. Consistent with other studies, in the present study carvone stimulated the cAMP pathway and increased the phosphorylation of Cdc25B and CDK1 via cAMP in conjunction with decreased proliferation of melanoma cells.

In the present study, we used B16F10 malignant melanoma cell line. Considering that several hyperpigmentation-related disorders such as melasmas and lentigines are non-malignant, it is not fully clear whether carvone also shows anti-pigmentation effects in the above-mentioned disorders. Further study is needed to confirm the beneficial effects of carvone in non-malignant melanocytes cell model.

It has been found that phytochemicals such as caryophyllene and isoorientin could reduce melanogenesis at the cellular level by down-regulating the expression of MITF and tyrosinase, the classical melanin production pathway [30,31]. On the other hand, our approach focuses on the inhibition of cell proliferation and the subsequent reduction of total melanin content. Considering these two strategies, a carvone-based approach could possibly be used alone or, desirably, in combination with other anti-melanogenic compounds for the treatment of pigmentation disorders.

We found that carvone treatment activated the cAMP signaling pathway (Figure 3), which is known to be upregulated in a relatively short time in response to various stimuli [32,33,34]. Although the mechanisms by which carvone raises cAMP concentrations are unknown, it is worth noting that carvone has been identified as a ligand of numerous olfactory receptors (ORs; e.g., OR1A1, OR8B3, and OR5P3, which constitute the largest subfamily of G-protein-coupled receptors) in each OR-transfected HEK293 cell line [35,36]. G-protein-coupled receptor activation in the membrane can effectively stimulate ADCY, which catalyzes the conversion of ATP to cAMP [37,38]. Further research is necessary to elucidate the exact mechanism by which carvone activates the cAMP signaling in melanoma cells.

It has been reported that the cAMP can stimulate melanogenesis by upregulating MITF and tyrosinase [39,40,41,42]. In the present study, carvone caused a modest but significant increase (121% vs. control) in cAMP levels; however, MITF and tyrosinase gene expression and melanin content were not significantly increased by the carvone treatment of B16F10 cells (Figure 1 and Figure 4). Although it is not entirely clear why melanogenesis was not induced during cAMP stimulation, we can hypothesize that the extent of the increase in intracellular cAMP levels may not be sufficient to induce melanin synthesis. In support of this notion, Friedmann et al. have found that a marked increase in cAMP levels (950% of a control), which can be achieved by irreversible and direct stimulation of ADCY, leads to a 10.9-fold rise of melanin content, whereas a relatively small increase in cAMP levels (260% of the control), resulting from melanocortin 1 receptor stimulation, increases melanin content by only 35% in melanoma cells during 7-day culture [43].

We revealed that carvone treatment inhibits the proliferation of B16F10 melanoma cells. On the other hand, it is noteworthy that carvone did not decrease the proliferation of other normal skin cells (epidermal keratinocytes and dermal fibroblasts, which are the predominant cells in skin tissue). Taken together, these findings suggest that carvone is likely to be involved in the homeostasis of cell growth. This idea is supported by a report indicating that carvone causes a significant decrease in the viability of abnormally proliferating breast cells without decreasing the viability of normally proliferating epithelial breast cells [44]. Similarly, carvone slows the growth of abnormally proliferating colon cells at a specific concentration, but at that dose carvone does not inhibit the growth of normally proliferating colonic epithelial cells [45].

## 4. Materials and Methods

### 4.1. Cell Culture

B16F10 melanocytes (American Type Culture Collection [ATCC], Manassas, VA, USA), Hs68 fibroblasts (ATCC), and HaCaT keratinocytes (AddexBio Technologies, San Diego, CA, USA) were cultured in high-glucose Dulbecco’s modified Eagle’s medium (Hyclone, Logan, UT, USA) supplemented with 10% of fetal bovine serum (Hyclone) and antibiotics (penicillin and streptomycin; Gibco, Grand Island, NE, USA) at 37 ° C in a humidified atmosphere (incubator) containing 5% of CO_2_ (Sanyo, Osaka, Japan).

### 4.2. A Melanin Quantification Assay

B16F10 cells (1 × 10^5^ cells/well) were cultured for 48 h in a 12-well plate with either vehicle (DMSO; Sigma, St. Louis, MO, USA) or various concentrations (50–200 µM) of carvone (Sigma; purity 97%). If needed, the cells were pretreated with either vehicle (DMSO) or 50 µM SQ22536 (cAMP inhibitor; Sigma) for 30 min before the carvone treatment. The cells were harvested by trypsinization followed by washing with phosphate-buffered saline (PBS; WelGENE, Daegu, Korea). Each cell sample was resuspended in 400 μL of 1 N NaOH containing 10% of DMSO and heated at 70 °C for 2 h. Melanin amounts were estimated by means of absorbance at 480 nm on a microplate reader (M200; Tecan, Männedorf, Switzerland). Throughout all experiments, the final DMSO concentration was less than 0.05%.

### 4.3. A Cell Proliferation/Cytotoxicity Assay

The rate of inhibition of cell proliferation was measured by two methods including direct cell counting and an MTT assay. B16F10 cells were cultured in a 24-well plate (6 × 10^4^ cells/well) for direct cell counting and in a 96-well plate (1 × 10^4^ cells/well) for the MTT assay and were treated with either vehicle or various concentrations of carvone for 48 h. The cells in the 24-well plate were trypsinized, resuspended in the growth medium, and stained with trypan blue (Invitrogen, Carlsbad, CA, USA). Viable cell numbers were determined using an automated cell counter, CELLOP^®^ (Small Machines Company, Seoul, Korea). Additionally, for the evaluation of cell cytotoxicity, the number of nonviable cells was also counted and the percentage of dead cells was calculated (CELLOP^®^). For the MTT assay, the cells were incubated with 60 µL of the MTT reagent (4 mg/mL in PBS) for 3 h at 37 °C to allow formazan crystals to form. After that, the medium was removed and 300 µL of DMSO was added into each well to dissolve the formazan crystals. The colored solution in each well was analyzed at 595 nm on the microplate reader. Furthermore, to determine whether the proliferation of Hs68 and HaCaT cells was also affected by carvone treatment the same method was employed as above.

### 4.4. Measurement of Intracellular cAMP Levels

B16F10 cells (6 × 10^4^ cells/well) were cultured in a 24-well plate and treated with either vehicle or 200 µM carvone for 5, 15, 30, or 60 min. After the removal of the medium, the cells were washed with PBS and incubated with 0.1 M hydrochloric acid for 5 min. The resultant whole-cell lysate was heated at 100 °C for 10 min, and intracellular cAMP concentration was measured with a cAMP enzyme-linked immunosorbent assay kit (Enzo, Plymouth Meeting, PA, USA) according to the manufacturer’s instructions. The cAMP levels were normalized to protein content determined by the Bradford Assay (Bio-Rad, Hertfordshire, UK).

### 4.5. Western Blotting

B16F10 cells (4 × 10^5^ cells/well) were incubated in a six-well plate and treated with either vehicle or 200 µM carvone. To elucidate possible cAMP-driven regulation of PKA, PCNA expression and of Cdc25B and CDK1 phosphorylation, the cells were pretreated with either vehicle or 50 µM SQ22536. Then, the cells were harvested and lysed in PROPREP lysis buffer (Intron, Seoul, Korea). The protein concentrations were determined by the Bradford method as described above. Equivalent amounts of protein (30 μg per lane) were separated by sodium dodecyl sulfate 12% polyacrylamide gel electrophoresis. The proteins were next transferred to a nitrocellulose membrane (Whatman, Dassel, Germany). The membranes were blocked with 5% bovine serum albumin (LPS Solution, Daejeon, Korea) and then probed with primary antibodies (Cell signaling, Hitchin, UK; 1:1000 dilution). All bands were visualized by means of peroxidase-conjugated secondary antibodies (Sigma; 1:5000 dilution) using an electrochemiluminescence detection reagent (Biomax, Seoul, Korea). Finally, the protein band images were detected using Light-capture (ATTO, Tokyo, Japan). Glyceraldehyde-3-phosphate dehydrogenase (GAPDH; Cell signaling; 1:5000 dilution) served as an internal control.

### 4.6. Quantitative PCR

B16F10 cells (4 × 10^5^ cells/well) were seeded in a six-well plate and incubated with either vehicle or 200 µM carvone. After that, total RNA was extracted from the cells using TRIzol (Invitrogen). The mRNA was reverse-transcribed into cDNA with SuperScript Ⅳ Reverse Transcriptase (Invitrogen). The quantitative PCRs were conducted in a 20 µL reaction mixture—containing 10 µL of SsoAdvanced™ Universal SYBR Green Supermix (Bio-Rad), 25 ng of a cDNA template, and 10 pmol of each primer—on a CFX Real-Time System (Bio-Rad). The thermal cycling conditions were as follows: 95 °C for 30s followed by 40cycles of 95 °C for 12 s and 59 °C for 20s. The threshold cycle (C_t_) of each sample was determined by the 2^(−ΔΔCt)^ method. *GAPDH* served as a housekeeping gene for normalization. Primer sequences used for each gene are listed in Table 1.

### 4.7. Statistical Analysis

All experiments in this study were performed in triplicate. Data were expressed as mean ± standard error of the mean (SEM) and were statistically analyzed in SPSS 25 software (SPSS; Chicago, IL, USA) using Student’s *t* test with significance set at * *p* < 0.05, ** *p* < 0.01, and *** *p* < 0.001.

## 5. Conclusions

We demonstrated that carvone markedly inhibits proliferation and subsequently reduces melanin content of melanoma cells via cAMP signaling. This study highlights the potential benefits of carvone for the treatment of melanomas and presumably other hyperpigmentation-related dermatological disorders such as melasmas, lentigines, and excessive freckles. Further study is required to confirm the potential of carvone as a functional ingredient for the cosmetics, food, and pharmaceutical industries.

## Figures and Tables

**Table 1:** Primer sequences.

Gene Description	Sequence (5′→3′)
Microphthalmia-associated transcription factor (MITF)	F: AGCGTGTATTTTCCCCACAG R: TAGCTCCTTAATGCGGTCGT
Tyrosinase	F: CCTCCTGGCAGATCATTTGT R: GGCAAATCCTTCCAGTGTGT
Glyceraldehyde-3-phosphate dehydrogenase (GAPDH)	F: GTGATGGCATGGACTGTGGT R: GGAGCCAAAAGGGTCATCAT

## Supplementary Materials

The following are available online at, Figure S1: Carvone has no influence on the proliferation of Hs68 dermal fibroblasts and HaCaT keratinocytes.
