# Supplementary material for: Carvone Decreases Melanin Content by Inhibiting Melanoma Cell Proliferation via the Cyclic Adenosine Monophosphate (cAMP) Pathway
Source: Molecules. 2020 Nov 7;25(21):5191. doi: 10.3390/molecules25215191 (PMC7664693; doi:10.3390/molecules25215191)
Supplement: Supplementary file 1 [file molecules-25-05191-s001.zip › Supplementary_Material (201104).docx]

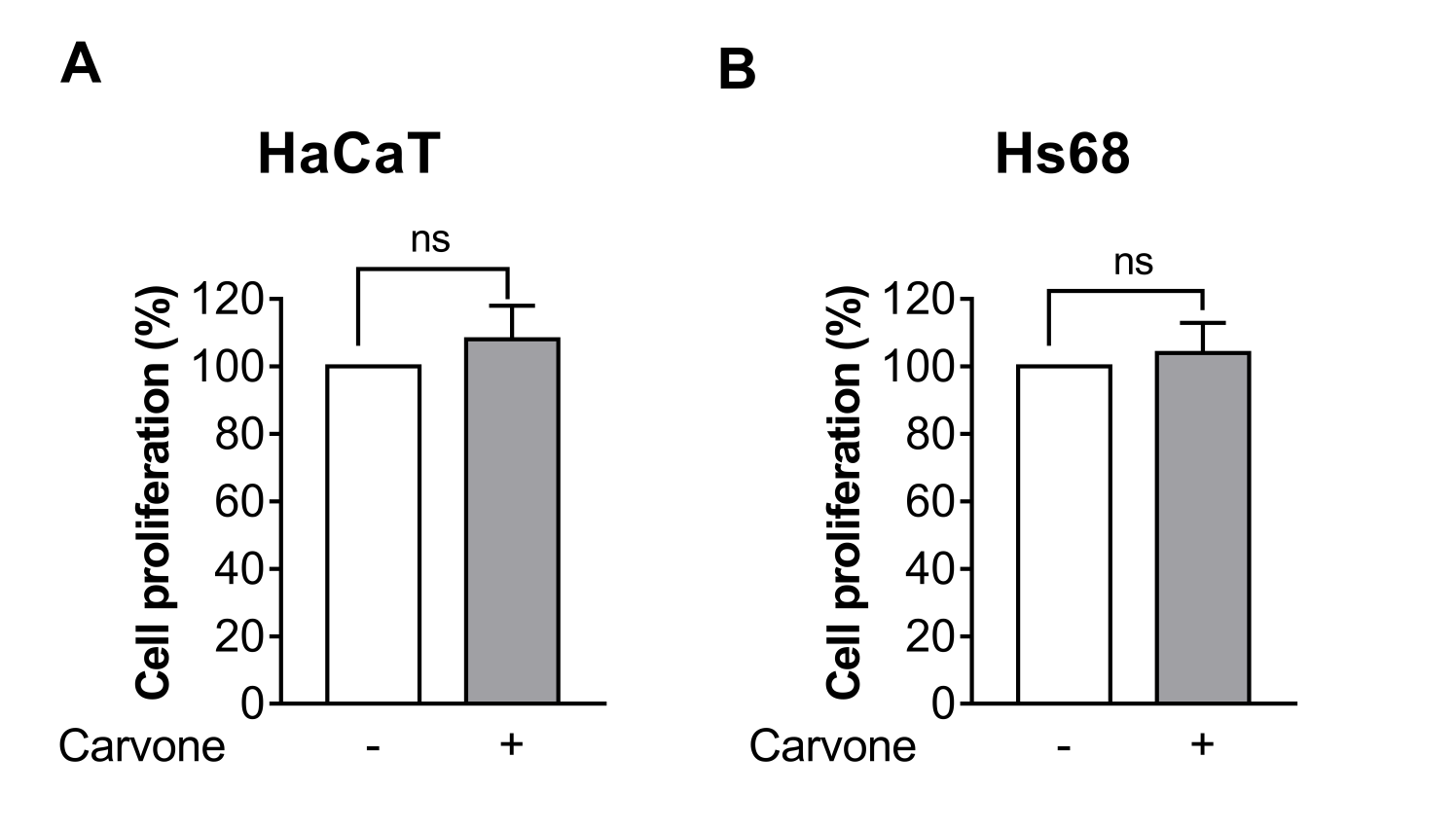


Figure S1. Carvone has no influence on the proliferation of Hs68 dermal fibroblasts and HaCaT keratinocytes. The cells were treated with carvone (200 µM) for 48 h. (A, B) The cell proliferation was quantified by the MTT assay. The data are presented as the mean ± SEM of three separate experiments.
