# Supplementary figures and images for: Carvone Decreases Melanin Content by Inhibiting Melanoma Cell Proliferation via the Cyclic Adenosine Monophosphate (cAMP) Pathway
Source: Molecules. 2020 Nov 7;25(21):5191. doi: 10.3390/molecules25215191 (PMC7664693; doi:10.3390/molecules25215191)

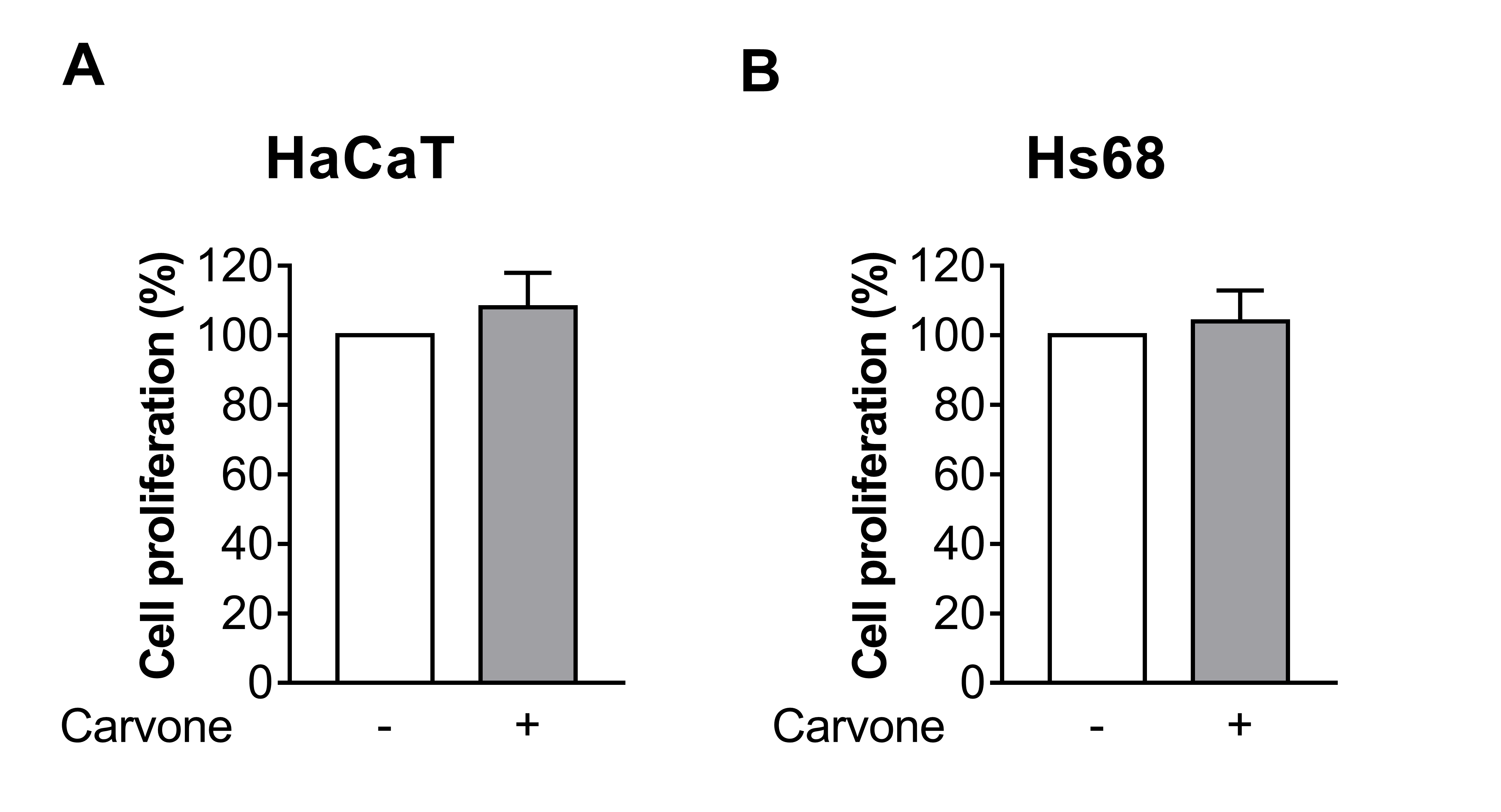

Supplement: Supplementary file 1 [file molecules-25-05191-s001.zip › Supplementary figure.tif]
